# Mental health around retirement: evidence of Ashenfelter’s dip

**DOI:** 10.1186/s41256-023-00320-3

**Published:** 2023-08-24

**Authors:** Thang T. Vo, Tran T. Phu-Duyen

**Affiliations:** 1 Health and Agricultural Policy Research Institute, 279 Nguyen Tri Phuong, District 10, 72406 Ho Chi Minh City, Vietnam; 2 School of Economics, University of Economics HCMC, 279 Nguyen Tri Phuong, District 10, 72406 Ho Chi Minh City, Vietnam

**Keywords:** Retirement, Mental health, Instrumental variable, SHARE, Panel data, Ashenfelter’s dip

## Abstract

**Background:**

Mental health issues among retirees have become increasingly concerning because the aging population presents a significant challenge globally, particularly in Western countries. Previous studies on this issue are plagued with bias owing to lacking panel data and estimation strategies. This study investigated the depression levels of European adults around the time of retirement.

**Methods:**

We used data obtained from Waves 1–7 of the Survey of Health, Ageing, and Retirement in Europe (SHARE) to create panel data covering the 2004–2017 period. Wave 3 (SHARELIFE) was excluded from the sample because it provided mismatched information. Fixed-effects (FE) and fixed-effects instrumental variables (FE-IV) models with multiple imputations were employed to examine the impacts of retirement on mental health before and after retirement, where being over pension age (normal and early) was used as the instrument variable.

**Results:**

Our results indicated that retirement based on aspirational motivations (β =  − 0.115, p < 0.001) and positive circumstances (β =  − 0.038, p < 0.001) significantly reduced depression, whereas retiring under negative circumstances could deteriorate one’s mental health (β = 0.087, p < 0.001). FE and FE-IV models indicated that overall, retiring reduced retirees’ depression (β =  − 0.096, p < 0.001 and β =  − 0.261, p < 0.001, respectively). The results of FE-IV models showed that adults planning to retire in the next two years experienced less depression compared with others in the workforce (λ =  − 0.313, p < 0.01). These adults must have adjusted their lifestyles in response to their impending retirement, thereby evincing Ashenfelter’s dip. Two years after retirement, when the “honeymoon” phase was over, retirees may have completely adapted to their new lives and the effect of retirement was no longer important.

**Conclusions:**

Retirement improves mental health before it happens, but not after. Increasing the pension eligibility age may postpone the beneficial effects of retirement on health. However, policy implications should be tailored according to the unique situations of each country, job sector, and population. Providing flexible schemes regarding retirement timing decisions would be better than a generalized retirement policy.

## Introduction

The effects of retirement on mental health constitute an issue of increasing concern because the aging population has become a significant challenge globally, particularly in Western countries [1–3]. Many European countries have increased the retirement ages and reduced the generosity of their pension systems [4] because increasing life expectancy and aging populations have placed enormous pressure on social welfare systems [5]. Although the pension system reforms discouraging early retirement can contribute to the sustainability of public finance, they may worsen the population’s quality of life because retirement is supposed to remove work-related stress and has an important influence on the mental health of the elderly [6]. Therefore, studying the impacts of retirement on mental health is necessary to shed light on retirement and population health policies.

Retirement may affect mental health through contrasting mechanisms. It could positively impact well-being through three channels. First, as older employees enter retirement, the relief from the stress associated with working and precarious working environments could improve their mental health [7, 18–20]. Second, retirees have more leisure time than non-retirees; therefore, they have more time to engage in physical activities including exercise, which could improve their physical and emotional health [19, 21], or they may sleep better [22]. Third, retirees likely have more time to make new voluntary connections to build networks of their peers and increase their social capital, thereby improving their mental health [12, 23–25]. Conversely, retirement involves major changes and can be a stressful event for many people [8, 26], thereby negatively affecting mental health through different channels. For example, retiring could worsen mental health because of the retiree’s loss of bonds with former colleagues [20] or the loss of work-related social contacts and participation in work-based events [19]. Moreover, having an occupation is often considered as the basic role of an individual in society; therefore, people can lose their sense of “self” after retirement, have less self-respect, and feel isolated, worsening their mental health [18]. Financially, retirement typically leads to a decline in regular income, which induces feelings of financial insecurity for those with limited savings or other financial resources when they retire [12, 19]. Retirement requires people to adapt to changes in the frequency and intensity of their activities [27]. Consequently, these lifestyle adjustments contribute to negative health outcomes, including depression [28].

Studies on this issue report conflicting results owing to different strategies [3, 7]. Many studies find that retirement positively influences mental health [3, 8–10]. In Europe, several studies show that retiring may impact the mental health of older adults [7, 11, 12]. However, the influence may differ depending on duration (short term versus long term) [12, 13], gender [7], and geographical areas [14]. Conversely, early retirement might be associated with anxiety and depressive disorders [15] or deteriorating general health [16]. A study in Australia confirmed the effect of retirement on psychological distress among men but not women [17].

Earlier studies suffer from biases because the retirement decision is not a random process. Studies using conventional approaches with cross-sectional data are likely influenced by unobserved individual heterogeneities that could affect both mental health and retirement decisions [11, 29–31]. Longitudinal studies using fixed- effects (FE) approaches are needed to rule out the presence of time-invariant confounding factors [28]; however, such studies may also be biased by the presence of time-variant factors [13, 18]. Further, poor health may trigger retirement decisions [32–35], thereby presenting a potential reverse causality problem [3, 12, 18]. Therefore, several studies have used the fixed-effects instrumental variables (FE-IV) approach for panel data [3, 6, 7, 18, 36, 37].

Furthermore, although the mental health effects of retirement may depend on the context, few studies have accounted for the various conditions of retirement, differences among those approaching and entering retirement, problems in adapting to retirement [8, 38], or impact of socioeconomic differences [19]. Thus, few studies have addressed the important roles of the reasons for retiring, retirement timing, and cultural context of the retirement transition [19]. Doing so is crucial because differences in the mental adaptations of retirees might be caused not only by the cessation of work but also by a combination of earlier life conditions, socioeconomic status, motivations for retiring [38], and employment histories [19]. The most noteworthy limitation observed in previous studies is that the potential effects of pending retirement on workers’ well-being actual retirement may have been underestimated [12, 18]. Because retirement is a predictable event for most individuals, they tend to adjust their behaviors in response to the various stages of retirement [39, 40].

Therefore, employees who are approaching retirement could already be experiencing changes in their mental health [18]. However, to our knowledge, whether the effect of retirement on mental health precedes retirement has not been confirmed in the literature. This “pre-impact” is similar to the “pre-program dip” that was first presented by Ashenfelter [41], often referred to as Ashenfelter’s dip, which describes the decline in outcomes the actual participation of an individual in a particular program. The influence of retirement on (mental) health may not occur immediately [42]. Instead, the well-being of the retirees could improve or worsen before the actual event [18]. Therefore, it is crucial to observe changes a few years before and after retirement [43]. In this study we investigated the depression levels of European adults around the time of retirement.

## Methods

### Study design

Acknowledging the limitations of previous research, we used panel data from the Survey of Health, Ageing and Retirement in Europe (SHARE) to investigate the mental health effects surrounding retirement among the European population. The FE models were employed to control for unobserved time-invariant confounding factors, and instrumental variables (IVs) were included to reduce potential biases from the endogeneity problem. Moreover, the reasons for retirement were included as explanatory variables. Notably, instrumental variables representing “predictive retirement” and “retirement in the past” allowed us to examine mental health effects before and after actual retirement, respectively, which distinguishes this study from others on this topic.

### Variables

#### Mental health

An individual’s mental health outcome was represented by the variable EURO-D, developed by the EURODEP Concerted Action Programme [52]. EURO-D has been used in many studies investigating the mental health of the European population [6, 30, 53–55]. EURO-D uses a 12-item scale to measure where an individual is positioned on a range of being depressed or not depressed. The interviews used to obtain the EURO-D values were conducted in the local language and included questions regarding depression, pessimism regarding the future, suicidal feelings, guilt, sleeping difficulties, levels of interest, fatigue, irritability, concentration, appetite changes, and sadness and enjoyment. Every “Yes” answer to the questions was coded “1” and every “No” was “0.” The scores were summed for each respondent, and the resulting EURO-D score ranged from 0 (the respondent is not depressed at all) to 12 (the respondent is very depressed) [56, 57]. The imputation technique was applied for the EURO-D variable where observations were missing (see details in section A).

#### Retirement status

The central explanatory variable in this analysis was the retirement status, which took the value of “1” for retirees and “0” for employed and unemployed people. According to [12], retirement status has three different definitions. First, nonretirees are employed, unemployed but looking for work, homemakers, or permanently ill or disabled. The second definition includes homemakers and permanently ill or disabled persons with retirees if they report no paid work during the previous month, and the third includes only those who report being either retired or employed. However, from the literature, Heller-Sahlgren’s third definition is the most common approach [1], which has been used in various studies (e.g., [1, 6, 13, 18]).

Using this definition is necessary for a meaningful inference. The current job situation according to SHARE data includes six groups: (1) retired, (2) employed or self-employed, (3) unemployed, (4) permanently sick or disabled, (5) homemaker, and (6) other. We excluded the unemployed, sick/disabled, homemakers, and others because comparing retirees with the employed would be coherent in terms of the discussion on the mechanism impacts of retirement, such as more available leisure time, reduction in work-related stress, reduced contact with former colleagues, and losing sense of self-worth (Table 1).

The lead retirement variable (retirement in the next wave(s)) took the retirement variable value in the next wave(s). For example, a person who would retired in the next wave had the “lead retirement 1” variable’s value of 1; a person retiring in the next 2 waves had the “lead retirement 2” variable’s value of 1. Conversely, the lag variable (retirement in the previous wave(s)) took the retirement value in the previous wave(s). Assuming that people did not return to work after retiring, missing variables were recorded as “1” if they retired in the last wave, and “0” if they did not retire in the next wave. A typical observation is illustrated in Table 2.

**Table 1:** Summary of all variables used in the models

Category	Variables	Values	Description
Dependent variable	Mental health: EURO-D	0–12	Range of being depressed. Higher values indicate higher depression
Independent variable	Retirement status	0–1	0: employed or unemployed 1: retired
Independent variable	Retirement lead 1/2/3	0–1	0: employed or unemployed 1: retired in 1/2/3 waves (2/4/6 years) later
Independent variable	Retirement lag 1/2/3	0–1	0: employed or unemployed 1: retired in 1/2/3 waves (2/4/6 years) before
Independent variable	Retired due to positive circumstances	0–1	0: employed or unemployed 1: retired because of at least one of the reasons: (1) became eligible for a public pension, (2) became eligible for a private occupational pension, (3) became eligible for a private pension, (4) received an offer of early retirement with special incentives
Independent variable	Retired due to negative circumstances	0–1	0: employed or unemployed 1: retired because of at least one of the reasons: lost their job due to redundancy (layoff), were in poor health, needed to care for an ill relative(s)/friend(s)
Independent variable	Retired due to aspirational motivations	0–1	0: employed or unemployed 1: retired because of at least one of the reasons: wanted to retire at the same time as their partner or spouse, wanted to spend more time with their family, or wanted to enjoy their life
Independent variable	Retired due to ambiguous reasons	0–1	0: employed or unemployed 1: retired with reasons fall into more than one category above
Instrumental variable	IV_normal	0–1	0: Not being over eligible pension age 1: Being over eligible pension age
Instrumental variable	IV_early	0–1	0: Not being over early pension age 1: Being over early pension age
Control variable	Gender	0–1	0: Female 1: Male
Control variable	Marital status	0–1	0: Not married 1: Married
Control variable	Vigorous activities	1, 2, 3, 4	Frequency of doing vigorous activities 1: More than once a week 2: Once a week 3: One to three times a month 4: Hardly ever, or never
Control variable	Moderate energy activities	1, 2, 3, 4	Frequency of doing moderate energy activities: 1: More than once a week 2: Once a week 3: One to three times a month 4: Hardly ever, or never
Control variable	Age	Integer (39–88)	Age at year of interview
Control variable	Number of children	Integer (0–19)	Number of children regardless of marital status
Control variable	GDP growth	Real	GDP growth of an individual’s country
	Unemployment rate	Real (0–100%)	Unemployment rate of an individual’s country

**Fig. 1:**
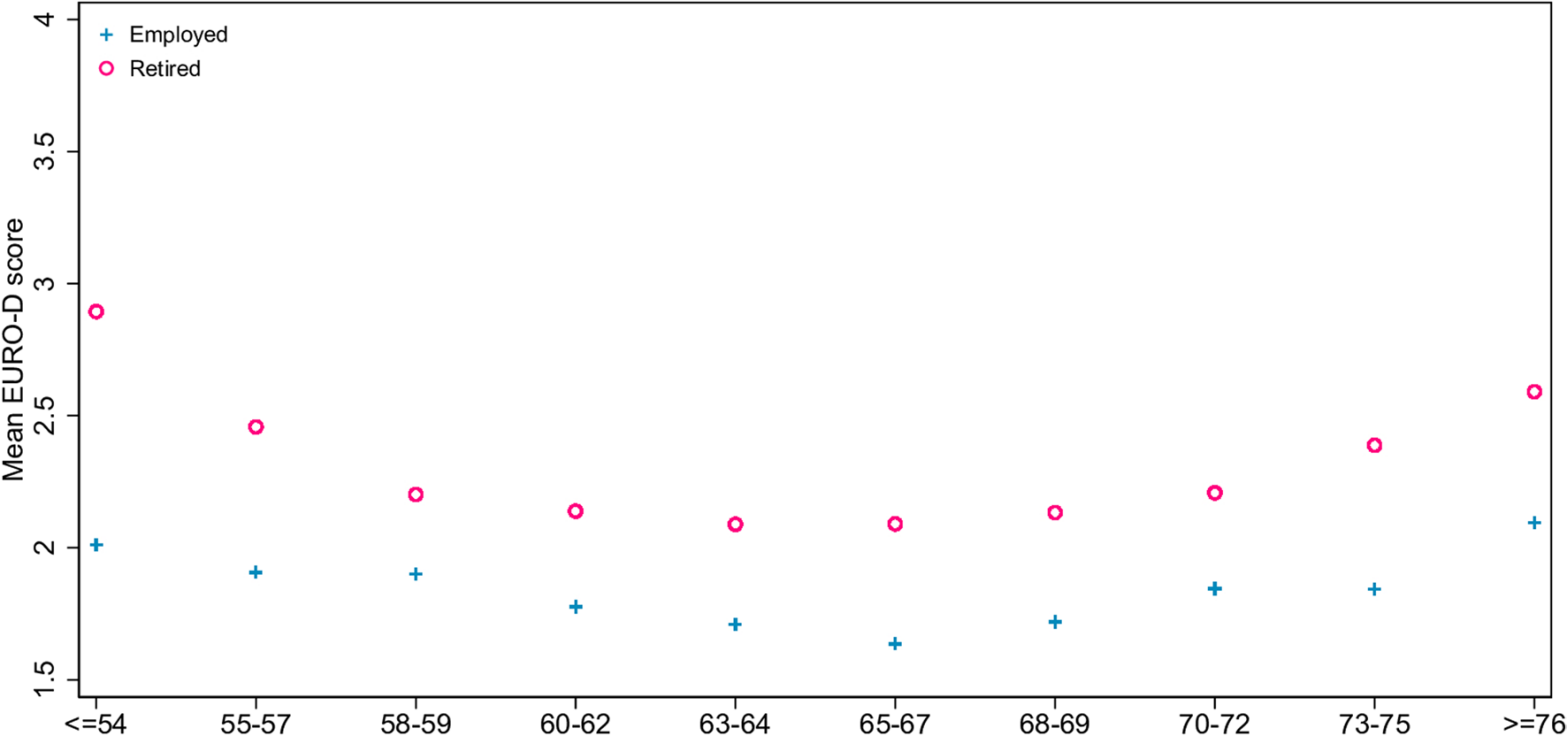
Observed depression level by age quantile

Figure 1 illustrates the depression levels of retirees and non-retirees by age. Figure 1 shows that the average levels of mental ill-health over time develop a U- shaped pattern, with the middle-aged and very old individuals having higher levels of depression. The dip in retirees’ depression was located at 63–64 years, whereas that of the employed was at 65–67 years. The mean eligible pension age for an individual in our sample was 64.4 years; Fig. 1 suggests that people, regardless of their retirement status, might feel less depressed when they approached retirement age but eventually became unhappy when they got older. In addition, retired people seemed to have more mental health problems than the employed. However, a relation between retirement and mental health cannot be posited because old age and retirement status are strongly related to each other.

#### Reasons for retirement

Ten reasons for retirement are listed in the SHARE data. The interviewees were asked whether they retired because they (1) became eligible for a public pension, (2) became eligible for a private occupational pension, (3) became eligible for a private pension, (4) received an offer of early retirement with special incentives, (5) lost their job due to redundancy (layoff), (6) were in poor health, (7) needed to care for an ill relative(s)/friend(s), (8) wanted to retire at the same time as their partner or spouse, (9) wanted to spend more time with their family, or (10) wanted to enjoy their life. However, we did not include each reason individually because there seem to be similar motivations across some reasons. Instead, we followed Robinson, who used 3 main reasons for retirement and divided the 10 SHARE reasons into 3 groups [58]. Reasons 1–4 formed the “positive circumstances” group; reasons 5–7 were included in the “negative circumstances” group; and reasons 8–10 were categorized as the “aspirational motivations” group. We classified respondents whose answers fell into more than one category as retiring due to “ambiguous reasons.”

#### Instrumental variables

Instrumental variables should meet two conditions: they are related to the explanatory variable and orthogonal to the exogeneity condition [3, 12]. Namely, retirement is the only “channel” through which the instrumental variables chosen in this study could alter the outcomes. Specifically, we followed studies that employed comparable models and chose the dummy of being past the eligible pension age and the dummy of being over the early pension age as the instruments [7, 30, 37].

#### Control variables

Control variables consisted of demographic background, activities, and survey dummies. First, the sociodemographic variables used were age and marital status [6, 7, 30, 42], number of children [30], and the country’s GDP and unemployment rate, which represent the countries’ economic background [59]. Adopting Zon and Butterworth’s method, marital status was divided into “married” and “not married” [1, 19]. The former category included people who were married and living with or without a spouse as well as those who had registered partnerships; the latter included people who were never married and those who were divorced or widowed. Additionally, because the act of taking care of other individuals may affect mental health, we considered the number of children as a variable in the models, regardless of whether they were living alone or with a spouse/partner. Second, activities were variables that represented the frequency of engaging in vigorous activities, such as sports or heavy housework, as well as activities that required moderate energy levels, such as gardening, car washing, or walking [7, 30]. Third, we used the dummies of SHARE’s waves to control for the time effects.

The summary statistics for the main variables are shown in Tables 3 and 4. The age of individuals in our data set ranged from 39 to 88 years, with an average of 65.07 years. The share of both genders was almost equal, 50.6% female and 49.4% male. Most of them were married; each person had an average of more than two children regardless of their marital status. For the frequency of engaging in physical activities, 73.2% of the people reported doing activities requiring moderate energy more than once a week. However, for heavy activities, they either infrequently did (more than once a week, 38.8%) or hardly ever or never did (36.5%) them. Non-retired individuals reported engaging in both kinds of activities more frequently.

**Table 2:** Retirement variables pattern

Mergeid	Waveid	Retirement	Lead 1	Lead 2	Lead 3	Lag 1	Lag 2	Lag 3
AT-020895–01	1	0	0	0	1	0	0	0
AT-020895–01	2	0	0	1	1	0	0	0
AT-020895–01	4	0	1	1	1	0	0	0
AT-020895–01	5	1	1	1	1	0	0	0
AT-020895–01	6	1	1	1	1	1	0	0
AT-020895–01	7	1	1	1	1	1	1	0

**Table 3:** Descriptive statistics of categorical variables by retirement status

Categorical variables	Not retired		Retired		Total	
	No	%	No	%	No	%
*Gender*						
Female	28,448	51.8	49,167	49.9	77,615	50.6
Male	26,487	48.2	49,433	50.1	75,920	49.4
Total	54,935	100.0	98,600	100.0	153,535	100.0
*Marital status*						
Not Married	11,817	21.7	26,056	26.7	37,873	24.9
Married	42,618	78.3	71,563	73.3	114,181	75.1
Total	54,435	100.0	97,619	100.0	152,054	100.0
*Vigorous activities*						
More than once a week	28,182	51.3	31,361	31.8	59,543	38.8
Once a week	8772	16.0	14,507	14.7	23,279	15.2
One to three times a month	4820	8.8	9824	10.0	14,644	9.5
Hardly ever, or never	13,140	23.9	42,871	43.5	56,011	36.5
Total	54,914	100.0	98,563	100.0	153,477	100.0
*Moderate energy activities*						
More than once a week	42,089	76.6	70,269	71.3	112,358	73.2
Once a week	7675	14.0	12,798	13.0	20,473	13.3
One to three times a month	2717	4.9	5266	5.3	7983	5.2
Hardly ever, or never	2433	4.4	10,242	10.4	12,675	8.3
Total	54,914	100.0	98,575	100.0	153,489	100.0

### Data source and processing

This research used data from the SHARE (see [57]), which is a longitudinal, multidisciplinary, and cross-national survey that collects data on the health and socioeconomic status of noninstitutionalized people aged over 50 years in 21 European countries and Israel, along with their social and family networks.

We extracted data obtained from Waves 1–7 of SHARE interviews and created panel data covering the 2004–2017 period. The Wave 3 and Wave 7 questionnaires contain SHARELIFE modules that focus on people’s life histories, including all the important aspects of respondents’ lives, but only Wave 7 has a regular panel questionnaire for all interviewees who previously answered SHARELIFE interview questions. Accordingly, we excluded Wave 3 because retrospective information was not considered in this study. The final unbalanced panel contains 182,142 observations from 6 survey rounds.

Being a large household survey, SHARE suffers from nonresponse issues [60], especially missing values [61]. SHARE release 7.0.0 provides five multiple imputations for the missing values of each variable [62]. Although there were few missing values of the variables used in our models (less than 5%), we accounted for them by using all five imputations from SHARE. Thus, all the variables in the analytical dataset came with five imputed values for each missing value. If a value is non-missing, the remaining associated imputed variables would have the same value as the base value. These variables in our data were imputed by SHARE’s hot-deck method. However, the estimated results were not much different from those of the dataset using single imputation or list-wise deletion (Details in Appendix 1).

As we exploited the longitudinal dimension of the SHARE database, the role of nonrandom attrition was concerning. Following Verbeek and Jones (see [63, 64]), we conducted two tests for attrition bias. First, we ran a regression of the depression score on an indicator counting the number of waves that an individual appeared in the panel. Second, we regressed the depression score on another indicator of whether an individual appeared in the next wave. Both regressions employed the pooled and random effect models with the unbalanced full sample, and the statistical significance of the two new indicators provided a test for nonresponse bias. In our study, all indicators’ coefficients were insignificant, thereby suggesting that there was no attrition bias (see Table 10 in Appendix 2). No matter how many times a respondent appeared in the panel, the EURO-D score was not systematically different. Other studies using the SHARE database have shown that nonrandom attrition was not an issue for the cognitive ability [65] or mental health represented by EURO-D [7].

### Statistical analysis

To investigate the possible correlation between retiring and one’s mental health, FE models were employed as shown in Eq. (1):

1 $$M{H}_{it}= \alpha +\beta Retir{e}_{it}+\delta {\overrightarrow{X}}_{it}+{u}_{i}+ {\in }_{it}$$

where *MH*_*it*_ and *Retire*_*it*_ denote a measure of mental health and retirement st0a1tus, respectively, of individual *i* at time *t*. ${\overrightarrow{X}}_{it}$ is a combination of control variables that represent the individual’s demographic background (age, marital status, and number of children) [1, 6, 7, 19, 30, 42] and factors that could affect the well-being of individuals, such as limitations regarding daily activities and the frequency of playing sports [7, 30]. Finally, *u*_*i*_ is the unobserved time-invariant heterogeneity with individual fixed effects, and *ϵ*_*it*_ represents distinctive error terms.

Coefficient *β* in the FE models is estimated under the assumption that *ϵ*_*it*_ is uncorrelated with *Retire*_*it*_ (the retirement decision). However, many researchers believe that this condition is easily violated by the presence of omitted variables and potential reverse causality, thereby causing endogeneity biases [3, 44]. Therefore, following [3, 7], and [12], we applied the FE-IV estimator to control for time-variant unobservable factors and reverse causal impacts.

2 $$Retir{e}_{it}={\theta }_{1}IVearl{y}_{it}+{\theta }_{2}IVnorma{l}_{it}+\delta {\overrightarrow{X}}_{it}+{u}_{i}+ {\in }_{it}$$

Equation (2) is the first stage of the FE-IV models, where *IV early*_*it*_ and *IV normal*_*it*_ are instruments for *Retire*_*it*_*.* Each instrument is defined as Instrument_*it*_ = $I(Ag{e}_{it}\ge Ag{e}_{t}^{p})$ where *I* is the indicator function, *Age*_*it*_ is the age of individual *i* at time *t* and *Age*^*p*^ is the country- and sex-specific pension age. We used both early and standard pension ages for each country. *I* takes the value “1” if the condition is true, and “0” otherwise. The second stage in the FE-IV estimation is shown in Eq. (3), which is similar to Eq. (1), wherein $\widehat{Retir{e}_{it}}$ is the predicted retirement status from the first stage function.

3 $$M{H}_{it}= \alpha +\beta \widehat{Retir{e}_{it}}+\delta {\overrightarrow{X}}_{it}+{u}_{i}+ {\in }_{it}$$

The coefficient β in Eq. (3) represents the average effect of retirement on mental health in the year of the survey. The results of this model are presented in the last two columns of Table 5. This impact may include effects from the current and past retirement. Therefore, we separated the impact of retirement in the past from the impact of current retirement by adding three *lags* of $Retir{e}_{it}$ to Eq. (3), indicating whether the individual retired in previous waves.

**Table 4:** Descriptive statistics of continuous variables

Continuous variables	N	Mean	Median	SD	Min	Max
*Eurod*						
Not retired	53,971	1.89	1.00	1.90	0.00	12.00
Retired	96,262	2.25	2.00	2.14	0.00	12.00
Total	150,233	2.12	2.00	2.06	0.00	12.00
*Age*						
Not retired	54,935	57.27	57.00	4.74	39.00	84.00
Retired	98,600	69.42	69.00	6.05	45.00	88.00
Total	153,535	65.07	65.00	8.09	39.00	88.00
*Number of child*						
Not retired	54,801	2.13	2.00	1.24	0.00	17.00
Retired	98,437	2.17	2.00	1.34	0.00	19.00
Total	153,238	2.16	2.00	1.30	0.00	19.00

4 $$M{H}_{it}=\,\alpha +\beta \widehat{Retir{e}_{it}}+{\gamma }_{1}{\widehat{Retire}}_{i\left(t-1\right)}+ {\gamma }_{2}{\widehat{Retire}}_{i\left(t-2\right)}+ {\gamma }_{3}{\widehat{Retire}}_{i\left(t-3\right)}+\delta {\overrightarrow{X}}_{it}+{u}_{i}+ {\in }_{it}$$

Similar to Eq. (3), the *lags* of $\widehat{Retir{e}_{it}}$ are instrumented by corresponding *lags* of early and normal IVs. For example, ${\widehat{Retire}}_{i\left(t-1\right)}$ is intrumented by $IVnorma{l}_{i\left(t-1\right)}$ and $IVearl{y}_{i\left(t-1\right)}.$ Thereby, the $\gamma$ coefficients show the impacts of past retirement on current mental health. In other words, they capture mental health impacts *after* retirement. Because the retirement event is predictable, the impact of the decision on mental health may not coincide with the exact retirement [18, 42]. Mental health may improve or decline in anticipation of retirement similar to Ashenfelter’s dip [41, 45–51]. Our study attempted to capture this potential effect by determining whether the level of mental health changed among those who knew they would retire in 2 years, 4 years, or 6 years compared with others in the workforce. The model is as follows:

5 $$M{H}_{it}=\,\alpha +\beta \widehat{Retir{e}_{it}}+{\lambda }_{1}{\widehat{Retire}}_{i\left(t+1\right)}+ {\lambda }_{2}{\widehat{Retire}}_{i\left(t+2\right)}+{\lambda }_{3}{\widehat{Retire}}_{i\left(t+3\right)}+\delta {\overrightarrow{X}}_{it}+{u}_{i}+ {\in }_{it}$$

where three *leads* of $\widehat{Retir{e}_{it}}$ denote whether an individual will retire in the next two years (or the next four or six years for the longer lead time). Estimations of the lead $\widehat{Retir{e}_{it}}$ are similar to those of the lag $\widehat{Retir{e}_{it}}$, using corresponding *leads* of early and normal IVs. The λ coefficients indicate the impacts of predictive retirement on current mental health. This model is expected to reveal the effect of retirement *before* it happens. We call this the “Ashenfelter’s dip” of mental health. Results of pre- and post-impacts are shown in Tables 6 and 7. All statistical and econometrical investigations were carried out with Stata software, version 17.

**Table 5:** Impacts of retirement and reasons of retirement on mental health

	FE		FE-IV	
	Without reasons	With reasons	Stage I	Stage II
Retired (*β*)	− 0.096 (0.022)***			− 0.261 (0.065)***
Retired due to aspirational motivations		− 0.115 (0.044)**		
Retired due to positive circumstances		− 0.038 (0.014)**		
Retired due to negative circumstances		0.087 (0.028)**		
Retired due to ambiguous reasons		− 0.005		
		(0.044)		
IV (normal pension age)			0.208 (0.003)***	
IV (early pension age)			0.224 (0.003)***	
Age	− 0.218 (0.019)***	− 0.225 (0.019)***	0.054 (0.003)***	− 0.218 (0.026)***
Age × Age	0.002 (0.000)***	0.002 (0.000)***	− 0.000 (0.000)***	0.001 (0.000)***
Marital status	− 0.460 (0.035)***	− 0.466 (0.035)***	0.015 (0.005)**	− 0.464 (0.036)***
Number of children	0.013	0.013	− 0.002	0.012
	(0.011)	(0.011)	(0.002)	(0.012)
Vigorous activities	0.045 (0.005)***	0.044 (0.005)***	0.004 (0.001)***	0.047 (0.005)***
Moderate activities	0.126 (0.007)***	0.128 (0.007)***	− 0.007 (0.001)***	0.127 (0.007)***
GDP growth	− 0.003	− 0.003	− 0.002	− 0.001
	(0.004)	(0.004)	(0.000)***	(0.004)
Unemployment rate	0.012 (0.002)***	0.012 (0.002)***	− 0.002 (0.000)***	0.010 (0.002)***
Constant	9.089 (0.954)***	9.345 (0.934)***	− 1.368 (0.178)***	9.835 (1.324)***
N	153,976	153,467	149,019	149,011

Standard errors in parentheses. *p < 0*.*05, **p < 0*.*01, ***p < 0*.*001

*Weak IV test*:

Under identification test by Anderson canon. corr. LM statistic: 1.1e + 04, *χ*^2^(1) P-val = 0.0000

Weak identification test by Cragg-Donald Wald F statistic: 6394.216

Stock-Yogo weak ID test critical values: 10% maximal IV size: 19.93, 15% maximal IV size: 11.59, 20% maximal IV size: 8.75, 25% maximal IV size: 7.25

**Table 6:** Pre-retirement impacts on mental health

	Stage I				Stage II
	Retired	Ret. 2 yrs later	Ret. 4 yrs later	Ret. 6 yrs later	
Corresponding IV*normal*	0.255 (0.003)***	0.122 (0.002)***	0.040 (0.002)***	0.016 (0.002)***	
Corresponding IV*early*	0.191 (0.003)***	0.166 (0.003)***	0.072 (0.002)***	0.011 (0.002)***	
Retired (*β*)					− 0.166 (0.071)*
Retired lead 1 (*λ*1)					− 0.313 (0.121)**
Retired lead 2 (*λ*2)					− 0.232
					(0.305)
Retired lead 3 (*λ*3)					− 2.116
					(1.249)
Age	0.021	0.023	0.016	− 0.000	− 0.187
	(0.003)***	(0.003)***	(0.002)***	(0.002)	(0.028)***
Age × Age	− 0.000 (0.000)***	− 0.000 (0.000)***	− 0.000 (0.000)***	− 0.000 (0.000)***	0.001 (0.000)**
Marital status	0.008	0.001	− 0.000	− 0.003	− 0.581
	(0.005)	(0.004)	(0.003)	(0.003)	(0.036)***
Number of children	− 0.003	0.000	− 0.001	0.001	0.019
	(0.002)*	(0.001)	(0.001)	(0.001)	(0.011)
Vigorous activities	0.002	0.001	0.000	0.001	0.051
	(0.001)***	(0.001)	(0.000)	(0.000)	(0.005)***
Moderate activities	− 0.006	− 0.002	0.000	− 0.000	0.135
	(0.001)***	(0.001)*	(0.001)	(0.001)	(0.007)***
GDP growth	− 0.001	− 0.001	0.002	0.000	− 0.000
	(0.000)*	(0.000)**	(0.000)***	(0.000)	(0.004)
Unemployment rate	− 0.006 (0.000)***	− 0.004 (0.000)***	− 0.002 (0.000)***	− 0.001 (0.000)***	0.005 (0.002)*
Constant	− 0.209	0.055	0.239	1.097	11.926
	(0.174)	(0.153)	(0.123)	(0.113)***	(1.650)***
F-test	3.68***	6.83***	12.71***	16.69***	
*Weak identification test*					
Cragg-Donald Wald F statistic	6394.216	3666.48	794.32	57.40	
Stock-Yogo weak ID test critical values					
10% maximal IV size	19.93	19.93	19.93	19.93	
15% maximal IV size	11.59	11.59	11.59	11.59	
20% maximal IV size	8.75	8.75	8.75	8.75	
25% maximal IV size	7.25	7.25	7.25	7.25	
*Weak-instrument-robust inference*					
Anderson-Rubin Wald test-F	10.38***	15.70***	13.93***	9.01***	
Anderson-Rubin Wald test-Chi-sq(2)	20.76***	31.41***	27.87***	18.03***	
N	175,722	175,722	175,722	175,722	173,510

Estimator: FE-IV. Standard errors in parentheses. *p < 0*.*05, **p < 0*.*01, ***p < 0*.*001

## Results

### Impacts of retirement and reasons of retirement on mental health

The estimations of the FE and FE-IV models with multiple imputations are presented in Table 5. The results the first column indicated that retirement made people feel less depressed by 0.096 points. The second column considered dummy variables for retirement reasons. First, people who retired because of aspirational motivations (e.g., to enjoy life, retire at the same time as their spouse, or have more time to spend with family) had better mental health than non-retirees (β = *−*0.115, p < 0.001). Second, retirees who stopped working because of positive circumstances (e.g., they became eligible to receive a pension or were offered an early retirement) might also experience lower levels of depression (β = *−*0.038, p < 0.001). Conversely, retirement due to negative circumstances deteriorated one’s mental health (β = 0.087, p < 0.001), but there was no clear evidence of changes in mental health among individuals who retired under ambiguous circumstances. The last two columns of Table 5 provided the results of the FE-IV models. Various tests to check the validity of the instrumental variables confirmed that the IV used in our study was appropriate (Results are presented in the footnotes of Table 5). The results for the first stage showed that being older than the standard or early pension age encouraged retiring decision-making (β = 0.208, p < 0.001 and β = 0.224, p < 0.001). The second stage of the FE-IV model indicated that retiring was likely to have a positive impact on the mental health of those eligible for retirement by 0.261 points (Column 4). This result was analogous to the result in the FE model. We tested the consistency of these models by applying the same specification with other datasets, which involve fewer waves. Similar results are presented in Tables 8 and 9 in Appendix 1.

**Table 7:** Post-retirement impacts on mental health

	Stage I				Stage II
	Retired	Ret. 2 yrs earlier	Ret. 4 yrs earlier	Ret. 6 yrs earlier	
Corresponding IV*normal*	0.255 (0.003)***	0.423 (0.003)***	0.652 (0.003)***	0.799 (0.003)***	
Corresponding IV*early*	0.191 (0.003)***	0.079 (0.003)***	− 0.073 (0.003)***	− 0.076 (0.003)***	
Retired (*β*)					− 0.305 (0.061)***
Retired lag 1 (*γ*1)					0.045
					(0.047)
Retired lag 2 (*γ*2)					− 0.025
					(0.035)
Retired lag 3 (*γ*3)					− 0.017
					(0.029)
Age	0.021	0.003	− 0.013	− 0.013	− 0.236
	(0.003)***	(0.003)	(0.003)***	(0.003)***	(0.024)***
Age × Age	− 0.000	− 0.000	− 0.000	− 0.000	0.001
	(0.000)***	(0.000)***	(0.000)**	(0.000)	(0.000)***
Marital status	0.008	0.011	0.013	0.010	− 0.574
	(0.005)	(0.005)*	(0.005)*	(0.005)	(0.035)***
Number of children	− 0.003	− 0.003	− 0.003	− 0.001	0.016
	(0.002)*	(0.002)	(0.002)*	(0.002)	(0.011)
Vigorous activities	0.002	0.000	− 0.001	− 0.001	0.050
	(0.001)***	(0.001)	(0.001)	(0.001)	(0.005)***
Moderate activities	− 0.006	− 0.001	− 0.000	0.001	0.136
	(0.001)***	(0.001)	(0.001)	(0.001)	(0.007)***
GDP growth	− 0.001	0.003	0.002	0.001	− 0.001
	(0.000)*	(0.001)***	(0.001)***	(0.000)**	(0.004)
Unemployment rate	− 0.006 (0.000)***	− 0.006 (0.000)***	− 0.005 (0.000)***	− 0.003 (0.000)***	0.009 (0.002)***
Constant	− 0.209	0.565	1.027	1.000	10.877
	(0.174)	(0.173)**	(0.174)***	(0.171)***	(1.228)***
F-test	3.68***	5.03***	4.47***	3.83***	
*Weak identification test*					
Cragg-Donald Wald F statistic	6394.216	13,399.69	25,430.88	34,772.69	
Stock-Yogo weak ID test critical values					
10% maximal IV size	19.93	19.93	19.93	19.93	
15% maximal IV size	11.59	11.59	11.59	11.59	
20% maximal IV size	8.75	8.75	8.75	8.75	
25% maximal IV size	7.25	7.25	7.25	7.25	
*Weak-instrument-robust inference*					
Anderson-Rubin Wald test-F	10.38***	14.04***	2.97	0.13	
Anderson-Rubin Wald test-Chi-sq(2)	20.76***	28.08***	5.93	0.27	
N	175,722	175,722	175,722	175,722	173,510

Estimator: FE-IV. Standard errors in parentheses. *p < 0*.*05, **p < 0*.*01, ***p < 0*.*001

### Pre- and post-retirement impacts on mental health

Tables 6 and 7 provide evidence regarding the mental health impacts before and after retirement, respectively. In the year of retirement, the effects were always significantly negative, thereby confirming that retirement improves mental health (β = *−*0.166, p < 0.05). Interestingly, the results of predictive retirement in the second stage indicated that only those who would retire in the next two years were less depressed than others in the workforce, whereas planning to retire in the next four or six years did not seem to have a significant impact on mental health (λ_1_ = *−*0.313, p < 0.01). The anticipation of the retirement event might have a stronger influence on mental health than the retirement itself. However, from two years after retirement, there was no evidence that retirement affects people’s mental health. In other words, the mental health of European retirees was not substantially different from that of non-retirees two years after retiring.

For other control variables, the models shared a common perspective. First, among those surveyed, younger individuals tended to report greater levels of depression. By growing older, these marginal effects shrank slightly. Being married reduced the retiree’s depression. The number of children they had did not seem to affect their mental health at all perhaps because the children of people at these ages were already grown up and independent from their parents. In addition, we replaced this variable with a dummy for having grandchildren, but it did not yield a significant result. Engaging in frequent physical activities considerably improved senior citizens’ mental health.

## Discussion

The results indicated that retirement decreased the levels of depression. Other studies have found similar results of the negative impacts of retirement on individuals’ mental health (e.g., [9, 38, 66]), including recent research based on SHARE data (e.g., [6, 7]). The finding that retirement due to positive reasons or “pull factors” leads to positive outcomes in psychological well-being has also been previously reported [67]. Interestingly, our results found that retirement improved the mental health of the elderly before retirement. These results implied that increasing the pension eligibility age may postpone the beneficial effects of retirement on health.

The impacts of retirement on mental health seem to vary, according to previous studies that applied comparable models. For instance, using SHARE data, Coe and Belloni found no significant correlation between the retired status and depression in their FE-IV models [7, 30]. For the UK population, Fleischmann found improvement in mental health before retirement and a steep improvement in the short term after that but not in the long term [68]. However, when Zhu used the same instrument in his FE-IV models with data from the Household, Income and Labor Dynamics in Australia Survey, he found the retirement status has a considerable impact on women’s mental health [3]. This result contrasts with the FE models used by the same author, which indicated that it might be the reverse causality of health on retirement decision in the FE models that produces these contrary results [3]. Moreover, using SHARE data (waves 1, 2 and 4), Heller-Sahlgren discovered a negative influence of retirement on depression levels in the long term [12]. Conversely, short-time effects were not confirmed with any statistical significance. Conflicting findings from the literature were explained by the differences in methodologies and countries studied [69].

The level of reported depression significantly reduced before retirement and increased shortly after retirement, which is consistent with other studies, as “retirement is seen as a process, rather than a one-time, one-way exit from the labor force” [70]. According to Atchley, whose retirement model is followed by most studies, retirement comprises six stages: preretirement, retirement, disenchantment, reorientation, retirement routine, and termination of retirement [71]. The first stage is often referred to as the “honeymoon” phase, wherein the retirees happily relish the free time and space they gain when they stop working [43, 72]. This is similar to the “growing interest in retirement” and “initial euphoria” stages of the five-stage model of [40]. In this phase, which is often six months, one year, or two years before retirement, preretirement self-esteem, positive friend identity meanings, and pension eligibility are the reasons for positive attitudes [73]. Moreover, the earlier stage of retirement can be occupied by hobbies, visiting, church activities, and traveling [74], which could lead to better mental health. In this study, because retirees changed their behaviors and lifestyles in anticipation of retirement, we considered the improvement in mental well-being before retirement as evidence of Ashenfelter’s dip.

Two years after retirement, the level of depression increased among retirees, which returned average depression to a “normal” level. This is similar to the results of Nyce, who found that retirement satisfaction decreases over time [75]. This phenomenon is reflected in Atchley’s “disenchantment” [71] or “stage with some stress” in Victor’s model [40]. In this phase, people tend to realize the reality of everyday life during retirement and eventually face emotional disappointment [43]. Subsequently, retirees adapt to their new lifestyles and become familiar with their new situations. They develop a realistic view of retirement and adjust accordingly in the last three stages of retirement [71].

One important policy implication of this topic is the determination of whether governments should adjust the retirement age. Increasing the pension eligibility age may postpone the beneficial effects of retirement on individuals’ health [69]. Increasing the retirement age for UK women by up to 6 years since 2010 has led to an increase in the probability of depressive symptoms [76]. However, to conclude that governments should postpone increasing the retirement age would be undesirable. Lowering the retirement age for Swiss construction workers was found to increase their self-reported health problems by 54% [77]. Furthermore, the impacts of retirement on health outcomes depends on multiple factors, including the characteristics of the job they retired from [68, 78, 79], lifestyle changes for retirement adjustment [80], personal perceptions of the retirement transition [81], and earlier life-course factors [82]. Therefore, policy implications should be contextual for each country, job sector, and particular population. In other words, providing flexible schemes for retirement timing decisions would be better than a generalized retirement policy.

Our paper has limitations because it did not consider the employment environment of the elderly before they retire or their perspectives regarding this major life event. Furthermore, there is potential for future investigation of retirees’ mental health. For instance, focus should be placed on issues of finance, housing, and jobs of the elderly approaching retirement and after that.

## Conclusions

This study investigated the depression levels of European adults around the time of their retirement. The findings indicated that retiring due to aspirational motivations and positive circumstances reduces the levels of depression, retiring under negative circumstances could escalate depression. Generally, the FE models indicated that retirement decreased the levels of depression. However, this result may include an upward bias because retirement is not completely exogenous to mental health. When instrumental variables were specified in the model, the results still confirmed that retirement significantly impacted mental health. The extended models showed that those who were going to retire in the next two years’ experienced lower levels of depression. These adults must have adjusted their lifestyles in response to their impending retirement. This particular impact before retirement is consistent with Ashenfelter’s dip. However, two years after the event, when the “honeymoon” phase was over, an increase in retirees’ depression brought their average mental health back to a normal level. From this point in time, the retirees gradually began to develop a realistic view of retirement and eventually adapted to their new lifestyles. At that time, the impact of retirement on mental health was no longer important. The findings in this study are supported by various models of retirement stages in the literature.

## Data Availability

The datasets supporting the conclusions of this article are included in additional files.

## Appendix 1: Multiple imputations

As a large household survey, SHARE suffers from nonresponse issues [60]. There are also missing values due to the survey design, such as branching, skip patterns, proxy interviews, country-specific deviations, and partial information [61, 62]. Hence, SHARE uses the hot-deck method and fully conditional specification method (FCS) to impute missing values of non-respondents. The FCS method is only employed for monetary variables, which are not used in our paper. However, hot-deck imputation method is conducted for several different types of variables with a small proportion of missing values (i.e., much less than 5%) [62] (Tables 8, 9).

**Table 8:** Impacts of retirement and reasons of retirement on mental health (Waves 2–4–5–6–7)

	FE		FE-IV	
	Without reasons	With reasons	Stage I	Stage II
IV*normal*			0.207 (0.0028)***	
IV*early*			0.200 (0.0032)***	
Retired (*β*)	− 0.106 (0.025)***			− 0.306 (0.077)***
Retired due to aspirational motivations		− 0.126 (0.052)*		
Retired due to positive circumstances		− 0.0326 (0.016)*		
Retired due to negative circumstances		0.117 (0.032)***		
Retired due to ambiguous reasons		0.0135		
		(0.051)		
Age	− 0.242 (0.023)***	− 0.253 (0.023)***	0.0642 (0.0042)***	− 0.236 (0.033)***
Age × Age	0.00169 (0.00011)***	0.00176 (0.00011)***	− 0.000441 (0.000016)***	0.00150 (0.00013)***
Marital status	− 0.474 (0.038)***	− 0.480 (0.038)***	0.0163 (0.0052)**	− 0.479 (0.039)***
Number of children	0.0102	0.0110	− 0.00286	0.00713
	(0.012)	(0.012)	(0.0016)	(0.012)
Vigorous activities	0.0425 (0.0052)***	0.0416 (0.0052)***	0.00388 (0.00069)***	0.0450 (0.0053)***
Moderate activities	0.122 (0.0073)***	0.123 (0.0075)***	− 0.00556 (0.00094)***	0.120 (0.0073)***
GDP growth	− 0.00724	− 0.00749	− 0.00237	− 0.00448
	(0.0038)	(0.0038)*	(0.00050)***	(0.0039)
Unemployment rate	0.0158 (0.0022)***	0.0158 (0.0022)***	− 0.00133 (0.00030)***	0.0147 (0.0023)***
Constant	10.39 (1.16)***	10.72 (1.13)***	− 1.917 (0.22)***	10.81 (1.75)***
N	140,850	140,392	136,878	136,870

Standard errors in parentheses. **p* < 0*.*05, ***p* < 0*.*01, ****p* < 0*.*001

**Table 9:** Impacts of retirement and reasons of retirement on mental health (Waves 4–5-6–7)

	FE		FE-IV	
	Without reasons	With reasons	Stage I	Stage II
IV*normal*			0.177 (0.0031)***	
IV*early*			0.149 (0.0035)***	
Retired (*β*)	− 0.0869 (0.030)**			− 0.409 (0.12)***
Retired due to aspirational motivations		− 0.137 (0.057)*		
Retired due to positive circumstances		− 0.0297		
		(0.017)		
Retired due to negative circumstances		0.110 (0.036)**		
Retired due to ambiguous reasons		0.0257		
		(0.056)		
Age	− 0.340 (0.048)***	− 0.344 (0.047)***	0.0836 (0.0058)***	− 0.288 (0.051)***
Age × Age	0.00177 (0.00018)***	0.00180 (0.00018)***	− 0.000481 (0.000022)***	0.00146 (0.00021)***
Marital status	− 0.536 (0.046)***	− 0.543 (0.046)***	0.0132 (0.0059)*	− 0.527 (0.047)***
Number of children	0.0158	0.0164	− 0.00148	0.0132
	(0.013)	(0.013)	(0.0016)	(0.013)
Vigorous activities	0.0367 (0.0058)***	0.0364 (0.0057)***	0.00361 (0.00070)***	0.0381 (0.0058)***
Moderate activities	0.104 (0.0081)***	0.105 (0.0083)***	− 0.00364 (0.00094)***	0.101 (0.0082)***
GDP growth	− 0.00928	− 0.00969	− 0.00359	− 0.00762
	(0.0041)*	(0.0040)*	(0.00048)***	(0.0041)
Unemployment rate	0.0181 (0.0037)***	0.0176 (0.0037)***	− 0.00306 (0.00044)***	0.0172 (0.0037)***
Constant	16.56 (2.80)***	16.63 (2.77)***	− 2.895 (0.33)***	14.72 (2.88)***
N	121,535	121,157	118,862	118,862

Standard errors in parentheses. **p* < 0*.*05, ***p* < 0*.*01, ****p* < 0*.*001

The hot-deck imputation method used in SHARE allows replacing the missing values of variables of a non-respondent (called the recipient) with the observed values from a “similar” respondent (called the donor). The donors are chosen randomly based on auxiliary variables which are also observed for the recipients. First, basic social-demographic variables such as age and education are imputed to be used as auxiliary variables to impute other variables. A set of auxiliary variables typically includes country, gender, age classes, groups of education years, and 2-scale self-reported health. Some variables require more auxiliary variables. For example, health-related variables are imputed based on those auxiliary variables plus an indicator for being hospitalized over last year [61, 62]. The SHARE release 7.0.0 provides five multiple imputations for missing values of each variable [62].

In this paper, we provide the main results with multiple imputations. We take advantage of all five imputations for all variables, including the main outcome EURO-D from SHARE, except for the two variables about vigorous and moderate energy activities. As SHARE does not provide imputations for those variables, we impute missing values of them by using age and gender as auxiliary variables (age and gender are the only variables without missing values in the data source).

## Appendix 2: Attrition

See Table 10.

**Table 10:** EURO-D and attrition

	(1) OLS	(2) RE	(3) OLS	(4) RE
Appear next wave dummy			* − *0*.*0641	* − *0*.*0420
			(0*.*043)	(0*.*036)
Appearance in panel				
1 times	0	0		
	(*.*)	(*.*)		
2 times	* − *0*.*0640	* − *0*.*0723		
	(0*.*076)	(0*.*080)		
3 times	* − *0*.*126	* − *0*.*139		
	(0*.*069)	(0*.*074)		
4 times	0*.*0857	0*.*0639		
	(0*.*100)	(0*.*13)		
5 times	* − *0*.*139	* − *0*.*161		
	(0*.*094)	(0*.*13)		
6 times (balanced panel)	* − *0*.*128	* − *0*.*158		
	(0*.*084)	(0*.*11)		
N	216,511	216,511	216,511	216,511

Standard errors are in parentheses. Control variables are suppressed

**p* < 0*.*05, ***p* < 0*.*01, ****p* < 0*.*001

## Abbreviations

SHARE: Survey of Health, Ageing and Retirement in Europe
EURO-D: 12-Item scale depression level
FE: Fixed effect(s)
IV(s): Instrument variable(s)
